# Enhanced Biocompatibility of Multi-Layered, 3D Bio-Printed Artificial Vessels Composed of Autologous Mesenchymal Stem Cells

**DOI:** 10.3390/polym12030538

**Published:** 2020-03-02

**Authors:** Eui Hwa Jang, Jung-Hwan Kim, Jun Hee Lee, Dae-Hyun Kim, Young-Nam Youn

**Affiliations:** 1 Division of Cardiovascular Surgery, Department of Thoracic and Cardiovascular Surgery, Severance Cardiovascular Hospital, Yonsei University College of Medicine, Seoul 03722, Korea; nurjih83@yuhs.ac (E.H.J.); JHKIM0907@yuhs.ac (J.-H.K.); VET1982@yuhs.ac (D.-H.K.); 2 Department of Nature-Inspired Nanoconvergence System, Korea institute of Machinery and Materials (KIMM), Daejeon 34103, Korea; meek@kimm.re.kr

**Keywords:** bioprinting, animal models, blood vessel, mesenchymal stem cells, growth factors

## Abstract

Artificial vessels capable of long-term patency are essential clinical tools in vascular surgery that involves small vessels. On-going attempts to develop artificial vessels that complements restenosis have not been entirely successful. Here, we report on the fabrication of small-sized artificial vessels using a three-dimensional bio-printer. The fabrication employed biodegradable polycaprolactone and autologous MSCs harvested from the bone-marrow of canines. The MSCs were cultured and differentiated into endothelial-like cells. After confirming differentiation, artificial vessels comprising three-layers were constructed and implanted into the arteries of canines. The autologous MSCs printed on artificial vessels (cell-derived group) maintained a 64.3% patency (9 of 14 grafts) compared with artificial vessels without cells (control group, 54.5% patency (6 of 11 grafts)). The cell-derived vessels (61.9 cells/mm^2^ ± 14.3) had more endothelial cells on their inner surfaces than the control vessels (21 cells/mm^2^ ± 11.3). Moreover, the control vessels showed acute inflammation on the porous structures of the implanted artificial vessels, whereas the cell-derived vessels exhibited fibrinous clots with little to no inflammation. We concluded that the minimal rejection of these artificial vessels by the immune system was due to the use of autologous MSCs. We anticipate that this study will be of value in the field of tissue-engineering in clinical practice.

## 1. Introduction

Cardiovascular surgery must often be accompanied by a vascular graft to replace or bypass dysfunctional blood vessels. These grafts may be autologous, in which case the saphenous vein and the internal mammary artery as the primary bypass vessel is often used, or they may be synthetic in origin, derived from polymers such as expanded polytetrafluoroethylene (ePTFE), Dacron, or polyethylene terephthalate (PET). However, the long-term maintenance of such grafts is limited due to complications from graft occlusion, thrombosis, rejection by the immune system, calcification, or infection [1]. Because of these limitations, alternative materials and methods with which to replace vascular grafts that are small in diameter are currently being explored. Specifically, methods using decellularization and scaffold fabrication with a variety of possible materials have been sought after. [2,3]. Decellularized vascular grafts have been developed in an attempt to prevent graft failure due to immune rejection. However, the acellular grafts are limited due to thrombosis upon exposure to blood and required additional procedures such as coating them with growth factors or vascular cell seeding [4]. Recently, cell-mixed hydrogels have been widely applied in medical engineering to promote cell fixation and the production of biological products for structural support and maintenance of cell functions [5]. Of the various hydrogel materials, alginate has been found to be particularly useful in the fabrication of hydrogels incorporating cells. When developing biocompatible materials using hydrogels such as alginates, controlling their viscosity and mechanical properties are important to preserving the viability of the cells [6,7]; although a high alginate content (high molecular weight) is advantageous in regards to cell fixation, it can cause apoptosis and a narrowing of vessel channels, which can interfere with cell proliferation [8]. In studies of vascular regeneration, an alginate content of less than 3% has been reported to maintain viable cells and promote the transport of substances such as vascular endothelial growth factor (VEGF), a potent signaling molecule that acts specifically in response to vascular endothelial cells. This suggests that an alginate-VEGF delivery system may be useful in vascular tissue engineering and wound healing applications [9]. 

In studies using 3D bio-printing with alginate solutions, 3% alginate was deemed an appropriate concentration with which to protect against physical denaturation [10], compared to 2.5% alginate, which, despite chemical crosslinking with CaCl2, facilitated poor formation of the designed pattern [10,11]. In addition to use in the cardiovascular system, constructing a double-structured conduit combining bio-printed alginate hydrogels and a biodegradable polycaprolactone (PCL) scaffold has been found to be beneficial in applications for treating the cranial nervous system and airway track: In the conduit in which alginate containing neurons and a porous PCL scaffold were combined, a uniform cell distribution and the same proliferation rate as that in plate media were confirmed [12]. Also, a bio-printed conduit manufactured with endothelial cell-containing, 3% alginate and a porous PCL structure was found to exhibit the expression of cells after application of a simulation system reflective of hemodynamics for 2 weeks [13]. Finally, the results of an artificial tracheal study showed that 3% alginate mixed with autologous epithelial and chondrocytes could be printed with PCL and support cell viability, showing dead cells at levels less than 20% from the third day [11]. Accordingly, we hypothesized that the fabrication of artificial vessels that are small in diameter with biodegradable PCL, an appropriate content of alginate, and a sufficient amount of autologous cells would protect against graft failure stemming from thrombosis, neo-intimal hyperplasia, and immune rejection.

The present study was undertaken to apply 3D bio-printing in the development of artificial vessels composed of biodegradable PCL, 3% alginate, and autologous mesenchymal stem cells (MSCs) holding the ability to differentiate into endothelial cells. Additionally, we assessed the biocompatibility of the artificial vessels in a canine animal model.

## 2. Materials and Methods

### 2.1. Scheme of Experimental Study Design

**Figure 1:**
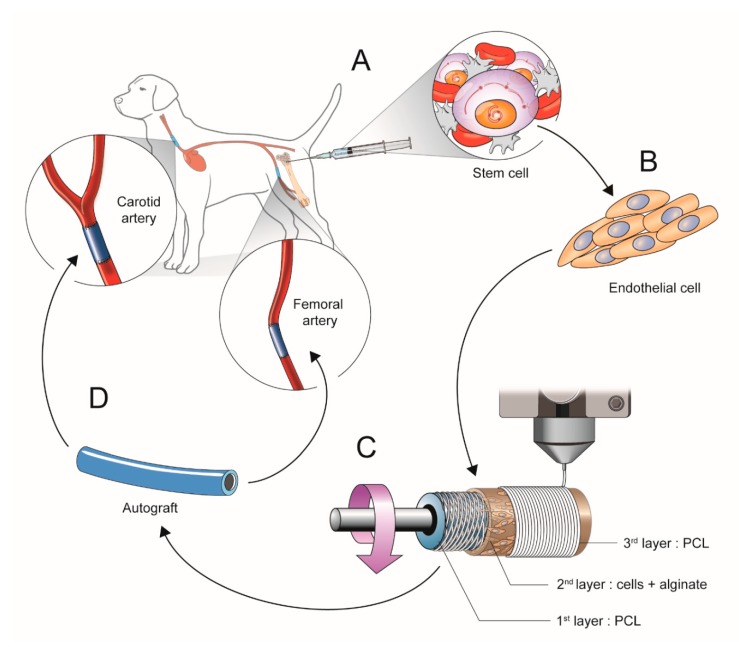
Scheme of the experimental study design. (**A**) The MSCs were cultured from bone marrow of canine femurs. (**B**) Cells resembling endothelial cells were differentiated for 7days. (**C**) The vascular scaffold was constructed with 3-layers (1st: PCL, 2nd: cells and alginate, 3rd: PCL) by 3D bio printing. (**D**) The vascular scaffold was implanted into the bilateral carotid arteries and femoral arteries of canines.

All experimental animal procedures and protocols were approved by the Animal Care and Use Committee of Yonsei University College of Medicine (2018-0092). An overview of the performed experiments is provided in Figure 1.

#### 2.1.1. Isolation and Culture of Mesenchymal Stem Cells (b-MSCs) from Canine Bone Marrow

In total, eight healthy Mongrel dogs with average weights of 33–38 kg were used in this study. Mongrel dogs anesthetized with an injection of intramuscular Zoletil (10 mg/kg) and Xylazine (5 mg/kg). Twenty milliliters of bone marrow was harvested from the femur of canines in heparinized conical tubes (SPL Life Sciences, Gyeonggi-do, Korea) and filtered through a 40-µm cell strainer (Life Sciences, Corning, NY, USA). After adding Phosphate buffered saline to adjust the volume of 8 mL, the mixture was transferred to a 15-mL conical tube containing 6 mL of Ficoll-Paque reagent (Sigma-Aldrich, Saint Louis, MO, USA) and centrifuged at 1840 rpm for 30 min. After centrifugation, b-MSCs isolated between the Ficoll-Paque reagent and blood plasma component. The aspirated b-MSCs were mixed with medium in 15-mL conical tubes. The cells in the medium were then centrifuged twice at 1500 rpm for 5 min and the supernatant was removed. Cells in the pellet were seeded on α-minimum essential medium (MEM, GIBCO, Grand Island, NY, USA) containing 10% fetal bovine serum (FBS, GE Healthcare Life Sciences, Pittsburgh, PA, USA), 1% penicillin-streptomycin (Thermo Fisher Scientific, Waltham, MA, USA), and 1% ITS+3 solution (Sigma-Aldrich, Saint Louis, MO, USA) at 37 °C in humidified air with 5% CO_2_. After attachment, the basal culture medium was changed every 2 days. The b-MSCs were cultured until passage three from the primary cell culture, when cell differentiation was assessed.

#### 2.1.2. Cell Differentiation into Cell Resembling Endothelial Cells

The b-MSCs were cultured in growth medium with 50 ng/mL of human VEGF (R&D Systems, Minneapolis, MN, USA) and 10 ng/mL of basic fibroblast growth factor (R&D Systems, Minneapolis, MN, USA) for 7 days. After 10 days, changes in cell morphology were observed under a phase-contrast microscope (Nikon, Sendai, Japan).

#### 2.1.3. Manufacturing the Artificial Vessels Using a 3D Bio-Printer

The small-diameter vascular scaffold was prepared with the MSCs (1 × 10^6^ cells/mL) and PCL with a molecular weight of 45,000 (Sigma-Aldrich, Saint Louis, MO, USA). The 3D printing device was supplied by KIMM & Protek Korea, (Yuseong-Gu, Daejeon, Korea). All procedures were performed under sterilized conditions, with sterile material. The vascular scaffold was designed to be 40 mm long and to have an inner diameter of 4 mm and an outer diameter of 5 mm [13]. 

The vascular scaffold structure comprised three layers. The first layer was constructed by diagonal cross-striping PCL through a 400-um nozzle at 110 °C, using a pneumatic pressure of 280 kPa and 20 rpm (pitch = 0.6 mm, feed = 800 mm/min). The second layer was constructed as a helix using the cell suspension, 3% sodium alginate and 1% calcium chloride solutions via a 300-um nozzle at room temperature (pitch = 0.5 mm, feed = 800 mm/min). The third layer was constructed in a helical form with PCL through a 400-um nozzle at 110 °C using a pneumatic pressure of 280 kPa and 40 rpm (pitch = 0.15 mm, feed = 800 mm/min). The bio-printed fabricated vascular scaffold of sodium alginate gel was fixed in 5% calcium chloride solution for 1 min to ensure that its bio-printed shape was maintained. It was then stored in an incubator in culture media without FBS at 37 °C in humidified air with 5% CO_2_.

#### 2.1.4. Surgical Implantation of the 3D Printed Vascular Grafts 

The small-diameter, artificial vessels were implanted into the bilateral carotid and femoral arteries of eight dogs. There were two experimental groups: the control group with vessels composed of PCL and alginate with no cells, and the cell-derived group with vessels composed of PCL and alginate with autologous cells. A total of 15 grafts (control group n = 7, cell-derived group n = 8) were implanted on both carotid arteries, and 10 grafts (control group n = 4, cell-derived group n = 6) were implanted on both femoral arteries in the eight dogs (Table 1). 

The bilateral carotid artery and femoral arteries were dissected through a midline incision. Heparin sodium (100 U/kg, Hanlim, Seoul, Korea) was administered before implantation of the vascular graft. End-to-end anastomosis was achieved using a 6-0 polypropylene continuous suture. After artery reperfusion, the absence of any leakage of blood flow on the interposition graft was confirmed, the incisional opening was closed, and the animal recovered. Aspirin (100 mg/day, BayerKorea, Seoul, Korea) and Clopidogrel Bisulfate (75 mg/day, Plavix, Sanofi Winthrop Industrie, Paris, France) were administered for 2 weeks. Sonography was performed 2 weeks after surgery. The implanted grafts were extracted for analysis and the animal was sacrificed under general anesthesia. 

### 2.2. Western Blot to Confirm Cell Differentiation

Proteins were extracted from cells after three passages with PRO-PREP reagent (Intron Biotechnology, Gyeonggi, Korea), incubated on ice, and centrifuged at 13,000 rpm at 4 °C for 5 min. The supernatant was then transferred to fresh tubes on ice. The protein concentration was analyzed with a Protein Assay Kit (Bio-Rad, Hercules, CA, USA). 

The proteins in samples containing 30 mg of protein were separated sequentially on 6% and 2% SDS/PAGE gels and transferred to a polyvinylidene fluoride membrane (Immun-Blot® PVDF Membrane, Bio-Rad, Hercules, CA, USA). Blotted membranes were blocked for 60 min with 3% BSA/5% skim milk and washed. The membrane was incubated overnight at 4 °C with each primary antibodies and 70 min at room temperature with each secondary antibodies. 

We employed α-smooth muscle actin as a marker for smooth muscle cells, von Willebrand factor and CD34 as markers for endothelial cells, and β-actin as a housekeeping protein. The following primary antibodies were acquired from Abcam: β-actin (mouse, 1:1,000, #8224), anti-CD34 antibody (rabbit, 1:1,000, #81289), anti-α-smooth muscle actin antibody (rabbit, 1:1,000, #5694), and anti-von Willebrand Factor antibody (rabbit, 1:500, #6994). The secondary antibodies were acquired from GEN Depot and were: goat anti-Mouse IgG (H+L)-HRP (1:10,000, #SA001-500) and goat anti-rabbit IgG-HRP (1:10,000, #SA002-500).

### 2.3. Scanning Electron Microscopy (SEM), Pre-Operation and Post-Operation. 

The artificial vascular grafts were examined by scanning electron microscopy (FE-SEM; Merin, Carl ZEISS, Oberkochen, Germany) both before and after implantation. Fixed specimens were dried and immersed in osmium solution (1% OsO_4_ in 0.1 M phosphate buffer) for 1.5 h. The dehydrated samples were coated with 5 nm of Pt with an ion coater (LEICA EM ACE600, Vienna, Austria). Samples were analyzed and photographed with a SEM (FE-SEM; Merin, Carl ZEISS, Oberkochen, Germany) at 2 Kv. 

### 2.4. Histological Assessment

Histological examination of the implants was performed at 2 weeks after implantation. Samples were fixed in 10% formalin and embedded in paraffin, after which sections of 5 µm thickness were taken. We evaluated the morphology of the vascularization and the structure of tubular samples stained with H&E using light microscopy. We also performed immunohistochemistry analysis for the presence of endothelial and smooth muscle cells. The primary antibodies, supplied by Abcam, were: anti-CD34 antibody (rabbit, 1:200, #81289), anti-α-smooth muscle actin antibody (rabbit, 1:100, #5694), and anti-von Willebrand factor antibody (rabbit, 1:1,000, #6994). The secondary antibody was anti-rabbit (Dako, k4003). Apoptosis was detected by the TdT-DAB and labeling (TUNEL) method using an in situ apoptosis detection kit (TREVIGEN 4810-30-K). Deparaffinized sections were incubated with TDT-DAB enzyme after treatment with proteinase K [14]. 

We evaluated inflammation with a scale defined as follows: a score of 0 was indicated no inflammatory cells surrounding the porous structure of the graft; a score of 1 indicated the presence of light, non-circumferential lympho-histiocytic infiltrate surrounding the porous structure of the graft; a score of 2 indicated the presence of localized, moderate to dense cellular aggregate surrounding the porous structure of the graft non-circumferentially; and a score of 3 indicated the presence of circumferential, dense, lympho-histiocytic cell infiltration of the porous structure of the graft. This inflammatory score was derived from an index for drug-eluting stents due to the lack of a similar index in the artificial vessel field [15,16,17]. Inflammatory scores were calculated as the sum of individual inflammatory scores for four zones in each section that was examined. 

Vascular endothelization was also assessed. The number of endothelial cells lining the inner surface of the lumen was calculated by determining the mean number of endothelial cells in the four zones in each section that was examined [18].

### 2.5. Statistical Analysis

Statistical analysis was performed with the SPSS software program (IBM, ver. 20). Quantitative data are expressed as mean ±SD. Probability values are two-sided from the Student’s *t*-test for continuous variables and the chi-square test for non-continuous variables. Statistical analyses of differences were performed using one-way analysis of variance (ANOVA) and Tukey’s multiple comparison tests. A value of *p* < 0.05 was considered statistically significant. 

## 3. Results

### 3.1. Validation of the b-MSCs Differentiation and Artificial Vessels

**Figure 2:**
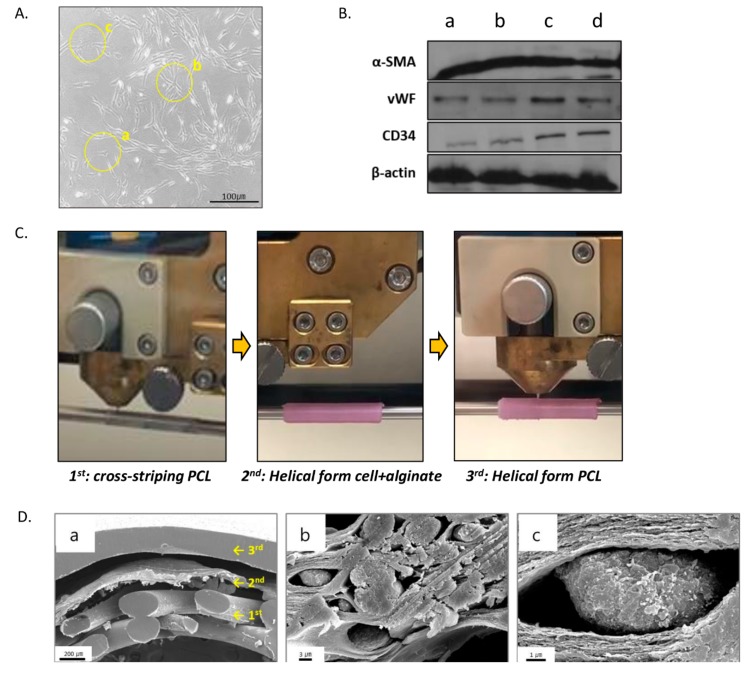
Validation of the materials and products (**A**) Morphology of the canine b-MSCs under a microscope (Nikon) (A-a) rapidly self-renewing cells, (A-b) spindle-shaped cells, and (A-c) flattened cells with nuclei. The original magnification of photomicrographs was X100. (**B**) b-MSCs differentiation in the cell-derived group (n = 4, a–d) was confirmed by Western blot analysis of respective differentiation markers; a-smooth muscle actin (α-SMA), von Willebrand Factor (vWF), CD34, and β-actin. (**C**) Prepared vascular scaffold by 3D printing (1st, cross-striping PCL; 2nd,^,^ helical form cell + alginate; 3rd, helical form PCL). (**D**) Cross-section SEM micrographs of pre-implanted artificial vessel by three-dimensional printing device. (D-a) Three layer artificial vessel (1st, porous structure PCL; 2nd, 1 × 10^6^ cells + 3% sodium alginate solution +1% cacl_2_; 3rd, non-porous PCL) (D-b) The middle layer combined with b-MSCs and alginate. (D-c) b-MSCs implanted between the alginate gel in the middle layer. Evaluated under original magnification for photomicrography. Magnification of A, B, and C was X40; X2.00K; X10.00K, respectively.

The b-MSCs were cultured from the primary cell culture until passage three, when cell differentiation was assessed. Cell differentiation was confirmed by morphological analysis and protein expression. We observed several different cell types and shapes under the microscope: rapidly self-renewing, star-shaped cells, (Figure 2A(a)), fibroblastic-like, spindle-shaped cells (Figure 2A(b)), and large, flattened cells with nuclei (Figure 2A(c)) [19]. We employed α-smooth muscle actin as a marker for smooth muscle cells, von Willebrand factor and CD34 as markers for endothelial cells, and β-actin as a housekeeping protein. These were detected by Western blot analysis (Figure 2B).

**Figure 3:**
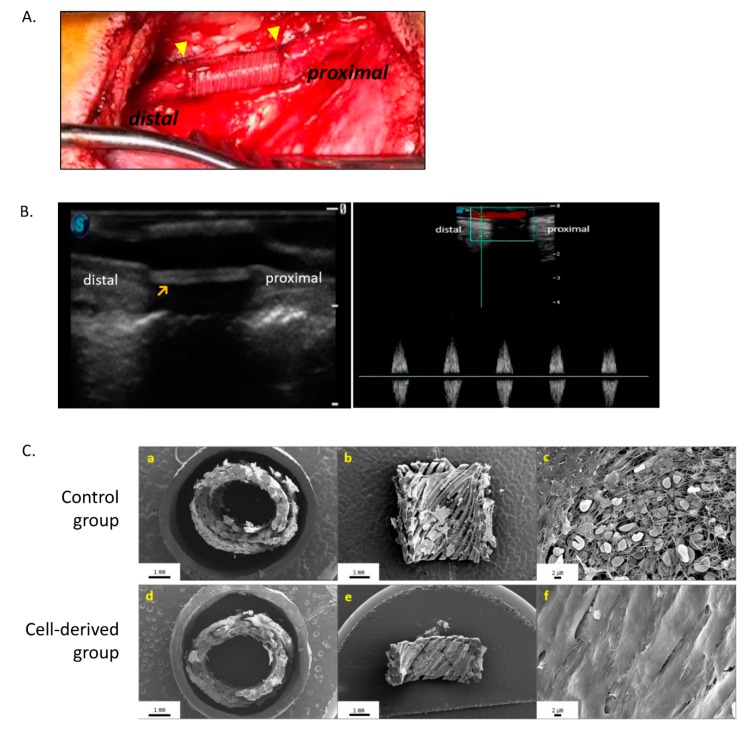
Implantation and assessment of the artificial vessels in a canine animal model (**A**) Intra-operative photography of end-to-end anastomosis between the graft and the carotid artery of the canine. (**B**) Doppler (left) and pulse Doppler flow (right) imaging of ultrasonography shows patent vessels at 2 weeks after implantation. (**C**) Cross- and longitudinal-section SEM micrographs of the artificial vessels post-implantation. (a–c) Thrombosis covered the inner surface of the artificial vessels in the control group. (d–f) Endothelial cells covered the inner surfaces of the artificial vessels in the cell-derived group. Evaluated under original magnification for photomicrography at X12 (a,d), X11 (b,e), and X2.00K (c,f).

We optimized the 3D-printed structure of the artificial vessels for prevention of the leakage of blood and exchange of substances with the blood. The optimal structure is shown in Figure 2C. To prepare the artificial vessels, we applied cells at a concentration of 1 × 10^6^ cells/mL and excluded FBS to prevent air bubbles. The artificial vessels were fixed in 5% CaCl_2_ for 1 min and incubated at 37 °C in humidified air with 5% CO_2_ overnight. Upon cross-sectioning and examining one of the vessels by electron microscopy, we detected the diagonal cross-striped pattern in the first layer and the fabricated helical forms in the second and third cell layers. The b-MSCs were embedded between the alginate gel (Figure 2D). The implants were then tested by implanting them into the carotid and femoral arteries of either a control group of dogs that received implants with no cells (control) or into an experimental group that received implants with cells (cell-derived) (Figure 3A). 

### 3.2. In Vivo Studies of 3D Bio-Printed Artificial Vessels

The patency of the implants at 2 weeks after implantation was defined as follows: (1) blood flow of the distal portion from the artificial vessels as assessed by Doppler ultrasound (Figure 3B), and (2) a patent area of 90% or more on histological images assessed with Image J software. Patency increased from 54.5% in the control group to 64.3% in the cell-derived group, although the difference was not significant (*p* = 0.422). Patencies for the individual arteries were 42.9% (control group) and 50.0% (cell-derived group) for the carotid arteries (*p* = 0.710) and 75.0% (control group) and 83.0% (cell-derived group) for the femoral arteries (*p* = 0.584). In all cases of occlusion, assessment of necropsy revealed thrombosis of the artificial vessels. 

**Figure 4:**
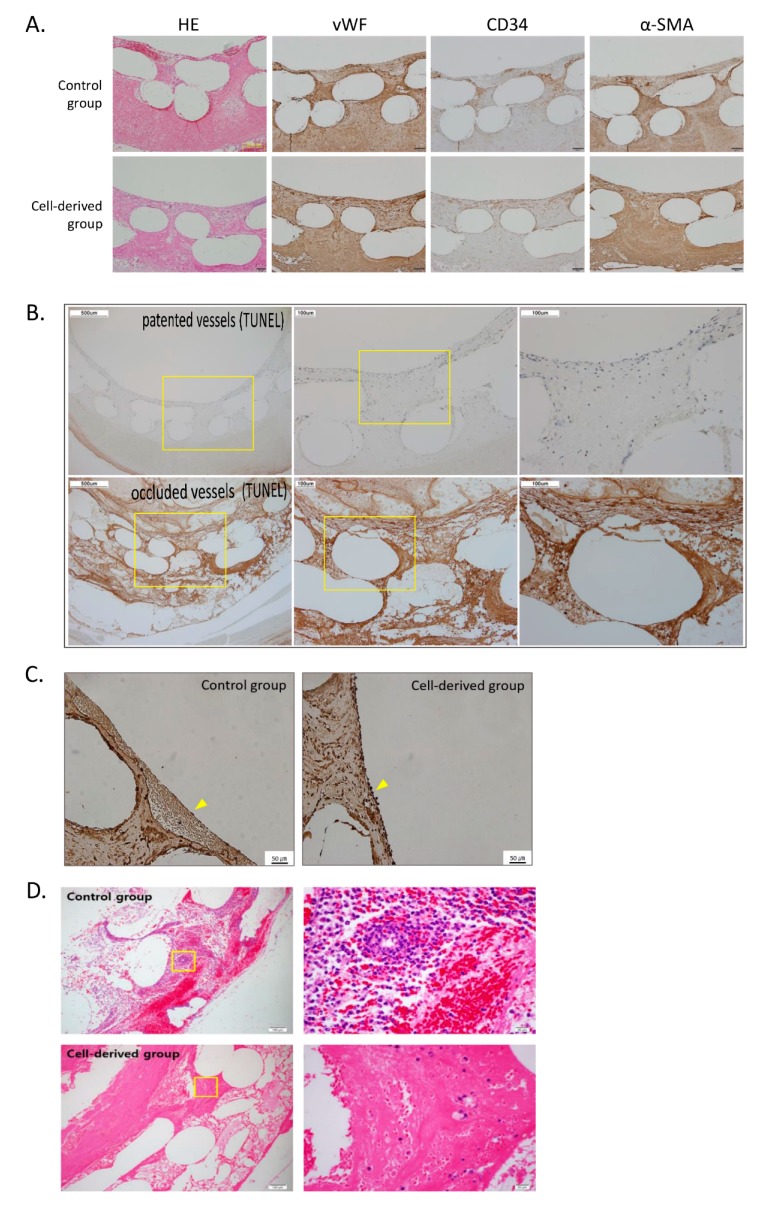
Pathology analysis of patented artificial vessels. (**A**) Implanted vessels were stained with H&E and IHC-P (vWF, CD34, α-SMA). Evaluated under an original magnification of X100 for photomicrography. (**B**) TUNEL staining between control group and cell-derived group. Evaulated under an original magnification of X40, X100, and X200. (**C**) Number of endothelial cells (ECs) was counted and comparatively analyzed on IHC-P (vWF) staining images using Image J software. Evaluated under an original magnification for photomicrography of x200. (**D**) The inflammation score was measured, and compared with the H&E staining image. Evaluated under an original magnification for photomicrography of X100, X600.

We conducted a comparative analysis of the patent vessels. We used their cross-sectional area to confirm their patency and assessed the inner surfaces of the vessels by SEM of longitudinal sections. The cell-derived vessels were surrounded by endothelial cells (Figure 3C(d–f)), while the inner walls of the control vessels were surrounded by thromboses (Figure 3C(a–c)). Staining with H&E revealed that control blood vessels were filled with thromboses (Figure 4A). Measurement of the expression of von Willebrand factor, CD34, and α-smooth muscle actin, confirmed our earlier observation that the cell derived vessels were surrounded by endothelial cells and contained smooth muscle cells (Figure 4A). 

The occluded and patented vessels of implanted cell-derived artificial vessels were compared and analyzed by TUNEL stain. TUNEL stain was expressed within occluded vessels, but revealed a few or no apoptotic cells within patented vessels among the transplanted artificial vessels (Figure 4B). 

The number of endothelial cells in the immunostained (vWF) images of the patent vessels were counted by Image J software. The results are shown in Figure 4C. There were significantly more endothelial cells in the cell-derived group than in the control group. Specifically, there were 61.9 cells/mm^2^ ± 14.3 in the cell-derived group and 21 cells/mm^2^ ± 11.3 in the control group (*p* = 0.012). Endothelial cells for the individual were 25.1 cells/mm^2^ ± 10.9 (control group) and 33.3 cells/mm^2^ ± 11.9 (cell-derived group) for the carotid arteries (*p* = 0.492) and 16.8 cell/ mm^2^ ± 11.8 (control group) and 84.7 cells/mm^2^ ± 16.2 (cell-derived group) for the femoral arteries (*p* = 0.013). When we viewed the implanted vessels under the microscope, we observed many acute inflammatory cell infiltrates in the control group, while they were only rarely observed in the cell-derived group. We assigned an inflammatory score to the porous structure of grafts to measure inflammation quantitatively, and these scores were significantly different between the groups: 1.4 ± 0.6 for the cell-derived group and 2.7 ± 0.6 for the control group (*p* < 0.001; Figure 4D). 

## 4. Discussion

We previously confirmed that pulsatile flow influences the fabrication of artificial blood vessels composed of differentiated cells resembling endothelial cells, alginate, and PCL, if applied at an appropriate time [13]. In this study, we produced and evaluated the function of artificial vessels small in diameter with differentiated cells, alginate, and PCL in vivo. We implanted the artificial vessels into dogs and confirmed that the inner luminal surfaces of the vessels were covered with endothelial cells and had little inflammation after implantation. 

Although various non-degradable materials are used in vascular replacement surgery, there are limitations to the uses thereof for fabricating vessels that are small in diameter because of thrombosis and the potential for blood clots [20]. Nontoxic biodegradable materials, such as PCL, are recommended when fabricating smaller artificial vessels [21]. PCL degrades slowly due to hydrolysis of its ester linkages, and the resultant fragments can be removed by macrophages and giant cells [20,22]. PCL, however, has poor hydrophilicity and low bioactivity [20]. In the present study, we have overcome these limitations by developing a porous structure that is covered with autologous mesenchymal stem cells. 

MSCs are phenotypically associated with vascular smooth muscle cells, and their pro-angiogenesis, immunosuppressive, anti-fibrotic, and anti-apoptotic properties in regeneration of damaged tissue have been demonstrated. MSCs also may inhibit the activation of natural killer cells, suppress dendritic cell maturation, change macrophages to an anti-inflammation phenotype, and modulate T helper 1 cells and TH17 cells to T helper 2 cells and regulatory T cells [23]. Additionally, several angiogenesis factors, such as VEGF, could be produced within MSCs, enabling vascularization within cells or tissues after implantation [23,24]. In a recent study, the authors noted that extracellular matrix was produced during a short period after implantation due to MSCs that had differentiated into endothelial cells, smooth muscle cells, and fibroblasts [18]. In the present study, we used endothelial cells that had differentiated from autologous MSCs to induce endothelization, to protect against apoptosis and immune responses, and to elicit the production of extracellular matrix of early after graft implantation. In histopathological comparisons, we observed that the number of endothelial cells increased as the implantation time increased, and we presume that contact with the blood flow through artificial vessels was a stimulator for arrangement of the endothelial cells (Figure 4C). As well, the differentiated ECs from the autologous MSCs were protected from immune response and apoptosis after graft implantation (Figure 4B,D) [25,26]. Also, the 3% alginate hydrogel may have played a role in promoting the differentiation of endothelial cells from the autologous MSCs and the exchange of materials for angiogenesis in the PCL structure. 

One concern we face is that the artificial autologous cell-derived vessels were not significantly more patent than the control vessels. It may be relevant here that canines are vulnerable to thrombosis and that the vessels in this study had quite small diameters (an inner diameter of 4 mm). Additional studies in other animal models, such as swine or rabbits, whose hematological characteristics are most similar to humans, might resolve this issue. We observed a significant difference between the groups in endothelialization and incidence of inflammation. Further studies, including long-term observations, should establish the clinical relevance and the long-term patency of these artificial vessels. Furthermore, artificial vessels could be a modified by the addition of specific cells and therapeutic agents to reduce the incidence of calcifications and to minimize the risk of rejection, infection, and thromboembolic complications.

## 5. Conclusions

In conclusion, we developed artificial vessels that were small in diameter using 3D bio-printing with 3% alginate hydrogel, PCL, and autologous MSC. Structurally, the first layer of cross-stripping PCL constructed to enable the exchange of materials to second layer of autologous MSC, and the third layer of helical form PCL constructed to protect blood leakage. Moreover, despite the small diameter artificial vessels, autologous MSCs elicited endothelialization on the inner luminal surfaces of the artificial vessels, and to protected against inflammation without acute thrombosis after graft bypass surgery.

## Figures and Tables

**Table 1:** The implanted vessels per position, and result.

Animal No.	Group	Position	Result
1	no cell (control)	left carotid artery (LCA)	damaged during dissection
		right carotid artery (RCA)	occlusion
		left femoral artery (LFA)	none implanted
		right femoral artery (RFA)	none implanted
2	no cell (control)	left carotid artery (LCA)	occlusion
		right carotid artery (RCA)	occlusion
		left femoral artery (LFA)	none implanted
		right femoral artery (RFA)	none implanted
3	no cell (control)	left carotid artery (LCA)	patent
		right carotid artery (RCA)	patent
		left femoral artery (LFA)	patent
		right femoral artery (RFA)	patnet
4	no cell (control)	left carotid artery (LCA)	occlusion
		right carotid artery (RCA)	patent
		left femoral artery (LFA)	patent
		right femoral artery (RFA)	occlusion
5	cell (cell-derived)	left carotid artery (LCA)	patent
		right carotid artery (RCA)	patent
		left femoral artery (LFA)	none implanted
		right femoral artery (RFA)	none implanted
6	cell (cell-derived)	left carotid artery (LCA)	patent
		right carotid artery (RCA)	patent
		left femoral artery (LFA)	patent
		right femoral artery (RFA)	patnet
7	cell (cell-derived)	left carotid artery (LCA)	occlusion
		right carotid artery (RCA)	occlusion
		left femoral artery (LFA)	patent
		right femoral artery (RFA)	patnet
8	cell (cell-derived)	left carotid artery (LCA)	occlusion
		right carotid artery (RCA)	occlusion
		left femoral artery (LFA)	patent
		right femoral artery (RFA)	occlusion
